# Prognostic Significance of Lymphovascular Invasion in Clinically Localized Prostate Cancer after Radical Prostatectomy

**DOI:** 10.1100/tsw.2008.49

**Published:** 2008-03-17

**Authors:** Dilek Ertoy Baydar, Barbaros Baseskioglu, Haluk Ozen, Pinar Ozdemir Geyik

**Affiliations:** ^1^ Department of Pathology, Hacettepe University, School of Medicine, Ankara, 06100, Turkey; ^2^ Department of Urology, Hacettepe University, School of Medicine, Ankara, 06100, Turkey; ^3^ Department of Biostatistics, Hacettepe University, School of Medicine, Ankara, 06100, Turkey

**Keywords:** prostate adenocarcinoma, lymphovascular invasion, prognosis

## Abstract

Whether lymphovascular invasion (LVI) is an independent prognostic factor in prostate cancer is still controversial. We retrospectively investigated its predictive role in disease progression following radical prostatectomy. The histological sections of radical prostatectomies from 71 clinically localized, prostatic adenocarcinoma patients were reviewed for LVI. Pre- and postoperative follow-up data were collected. LVI was identified in 15.5% of cases. Univariate analysis showed a significant association between LVI and advanced pathological stage, higher Gleason score, positive surgical margins, extraprostatic extension, seminal vesicle invasion, and lymph node metastasis (each *p* < 0.05). Multivariate analyses pointed to vascular involvement as a strong and independent predictor for PSA failure (*p* = 0.023), and reduced biochemical progression-free survival (*p* = 0.019). LVI in radical prostatectomy is an adverse prognostic finding that must be recorded in the pathology report.

## INTRODUCTION

Prostate carcinoma is a heterogeneous disease and its prognosis is highly variable. Many researchers focus on investigating significant markers of prognostic significance. The association of vascular invasion with unfavorable outcome has been known for a long time. However, the question is still whether or not it adds more beyond other commonly used standard parameters. Not all studies have found independent significance of lymphovascular invasion (LVI) in multivariant analysis. The Association of Directors of Anatomic and Surgical Pathology and the College American Pathologists (CAP) require that LVI is reported in transrectal needle biopsies or transurethral resection of prostate; however, reporting it on radical prostatectomy is not addressed[1,2]. On the other hand, the Association of Anatomic and Surgical Pathology recently assembled and updated the CAP protocol to comply with the American College of Surgery Commission on Cancer (COC) requirements[3]. In their checklist, a record of angiolymphatic invasion is required in pathology reports for radical prostatectomy.

Studies that issue the role of vascular invasion in prostate carcinoma are few. The reported incidence of LVI varies widely from 5 to 53%[4,5,6,7,8,9,10,11,12,13,14,15,16,17]. This wide range possibly results from difficulty in its recognition, deficiency of standardized histologic criteria, and variations in the study population. In a recent article, Cheng et al. showed a significant association between LVI and adverse pathologic features, including advanced pathological stage, higher Gleason score, positive surgical margins, extraprostatic extension, seminal vesicle invasion, lymph node metastasis, and perineural invasion, as well as higher preoperative serum prostate specific antigen (PSA)[15]. They determined LVI as an independent predictor of PSA and cancer-specific survival. The aim of our study is to investigate the prevalence and role of LVI as a prognostic indicator in our series of clinically localized, prostate carcinoma treated with radical prostatectomy.

## METHODS

Seventy-one clinically localized, prostate cancer patients treated with radical prostatectomy (RP) were included in this study. The RPs were done by one surgeon at the same academic institution between 1992 and 2001. Sixty-nine cases had pelvic lymphadenectomy. None of the patients had received adjuvant antitumor therapy before surgery. Pre- and postoperative data were collected on all patients from the hospital charts. Clinical follow-up data were available on 67 cases. The patients were postoperatively followed up for an average of 65 months (median, 54 months; range, 4–145 months). The serum levels of PSA were determined at 3-month intervals after RP for the first year and at 6-month intervals thereafter. Biochemical failure was determined by a constant or increasing PSA level of >0.2 ng/ml on two successive measurements.

All slides for each case were retrieved from the pathology archives and were reviewed by a single pathologist (D.E.B.). Prognostically well-established pathological parameters, including Gleason score, status of extraprostatic extension, seminal vesicle invasion, surgical margins, lymph node metastases, and pathologic stage (pT), in addition to the presence of LVI, were noted. Tumor volume was estimated by simple eye-balling. In the evaluation of vascular involvement, care was taken to exclude artifacts due to retraction or mechanical displacement of tumor cells into lumen by blade (pseudoemboli), tumor within prostatic ducts and within perineural spaces. For the equivocal cases, LVI was confirmed for the presence of endothelium using antibody to CD31 (Clone CJ70A, Dako, Copenhagen, Denmark). Differentiating blood vessels from lymphatics was not attempted since, for practical purposes, either lymphatic or venous involvement implies ultimate reach to the circulatory system.

Statistical analyses between groups were performed using Fisher's exact and chi-square tests for categorical variables and the Mann-Whitney U-test for continuous variables. Survival analyses, including time to PSA relapse or biochemical progression-free survival (PFS), were performed with Kaplan-Meier methods. Logistic regression and Cox proportional hazard models were used to carry out multivariate tests; $p \leq 0.05$ was taken to indicate statistical significance.

## RESULTS

Ages of the patients varied between 48 and 75 (average, 62; median, 63). PSAs at diagnosis ranged from 1.3 to 41.5 ng/ml (mean, 11.5 ng/ml). Pelvic lymph node metastasis was detected in five patients (one lymph node in two, two lymph nodes in two, and three lymph nodes in one patient). At a mean of 41 months after surgery, 22 cases had biochemical progression. Among them, eight patients experienced local recurrence of the tumor; one man developed distant metastases and died of the disease.

LVI was identified in 15.5% of patients (11/71). It was seen intratumoral in 6/11, at the periphery of the tumor in 2/11, both intra- and peritumoral in 1/11, and at a distant site from the tumor in 2/11 cases (Fig. 1). Average number of foci was 6.

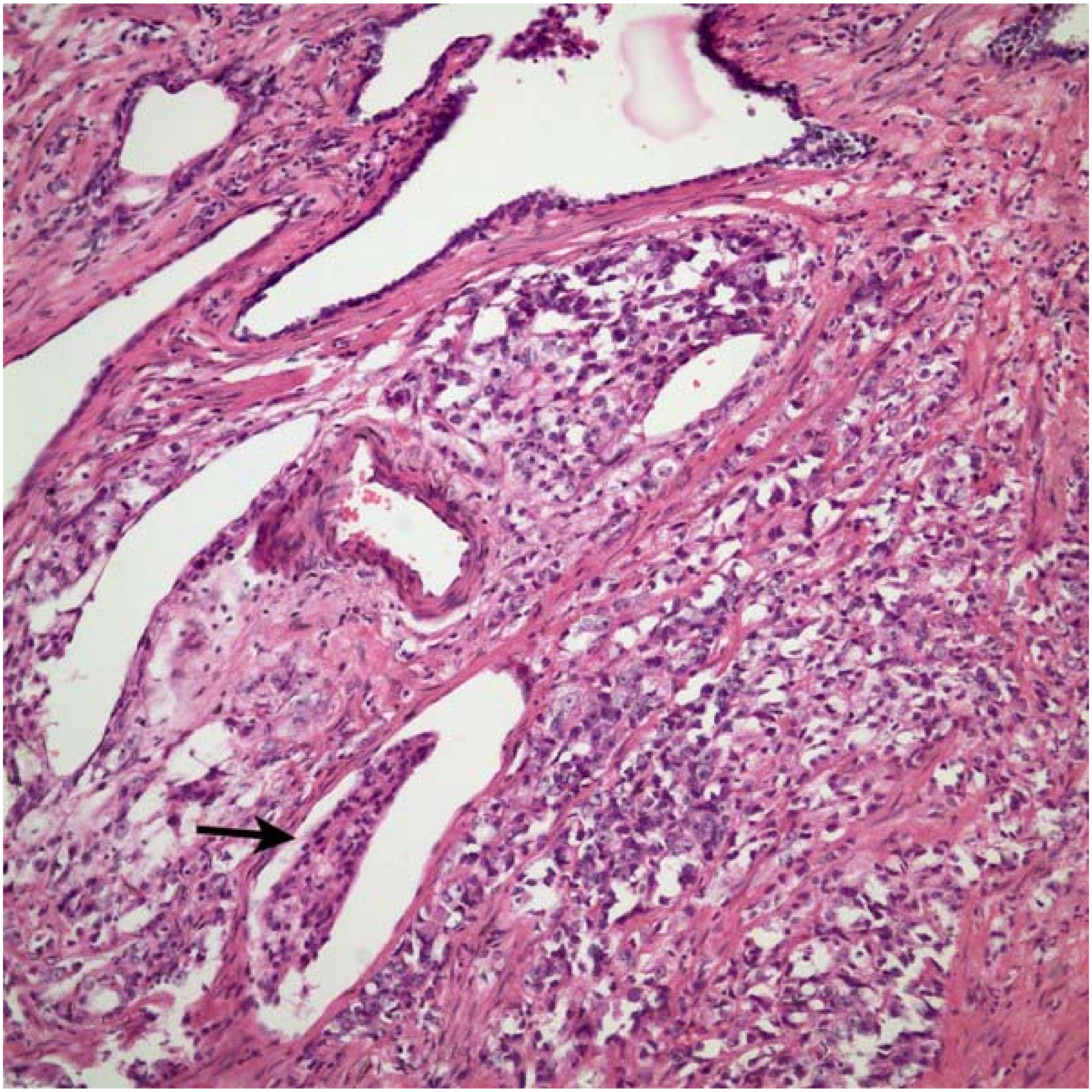

**A**

**FIGURE 1.** Intravascular neoplastic cells. (A) Inside the tumor (H-E ×400); (B) at the periphery of the tumor (H-E ×200); (C) distant from the tumor (H-E ×400).

Characteristics of the patients with LVI were summarized in Table 1. Age and preoperative PSA level did not differ between LVI-positive and -negative groups. A significant association was found between LVI with higher Gleason score, extracapsular tumor extension, positive surgical margins, seminal vesicle invasion, pathological stage, and lymph node metastases on univariate analyses (all $p < 0.05$) (Table 2) (Figs. 2 and 3).

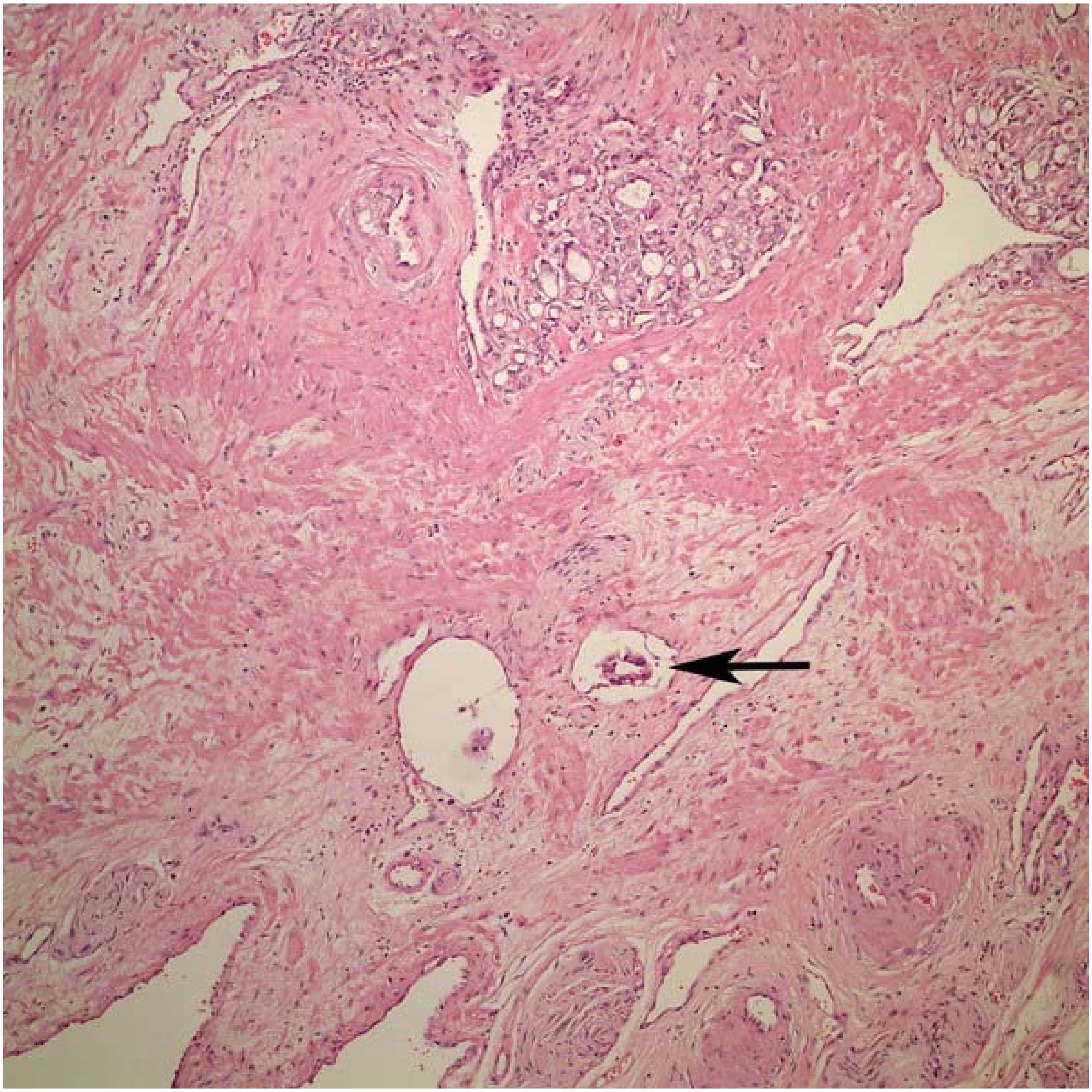

**FIGURE 1B**

Among 11 patients with LVI, 10 had follow-up data. Nine out of 10 had recurrent disease (seven isolated biochemical failure, two local relapse, and one distant metastasis). LVI was the strong independent predictor for PSA failure in multivariant analysis when taken into account with grade, pT, lymph node involvement, age at diagnosis, preoperative PSA levels, and positive margins ($p = 0.023$, logistic regression model). Gleason score was the other equally powerful independent variable of recurrence ($p = 0.023$).

In Cox regression analysis, LVI, pathological stage, and Gleason sum were the factors that determined the time to PSA failure (significance values 0.019, 0.027, and 0.049, respectively). The average time to biochemical PFS was significantly shorter for patients with LVI compared with patients without LVI (46.5 vs. 79 months, respectively). At 5 years, the PFS rate was 70% for patients without LVI, compared with 20% for patients with LVI (Fig. 4).

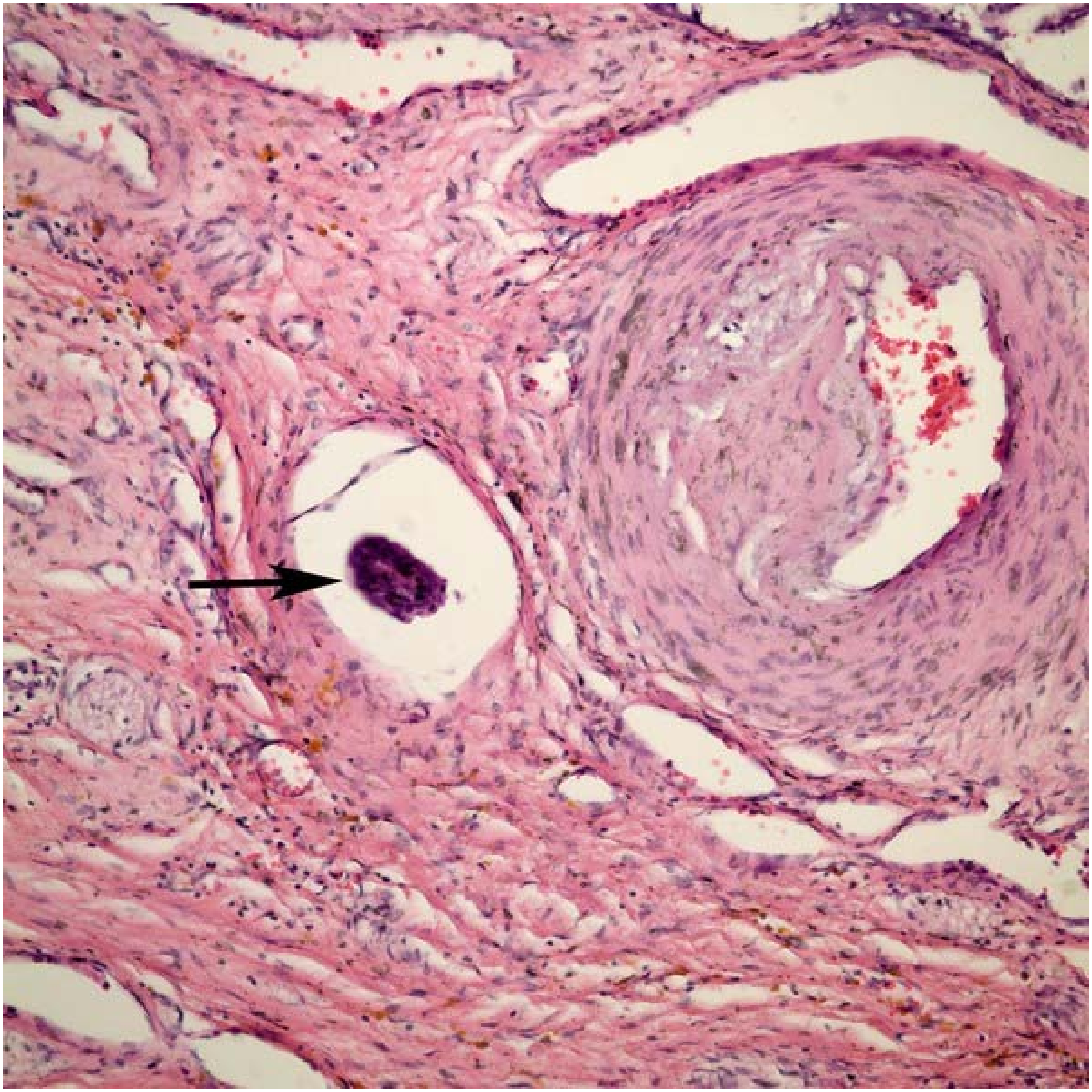

**FIGURE 1C**

## DISCUSSION

Pathology has assumed an important role in the assessment of adenocarcinomas of the prostate during the last years. Postoperative data from the pathologic workup of RPs, such as tumor histology, differentiation, volume, pathological stage, margin, and lymph node status, give important information for the prediction of tumor outcome. The weight of evidence suggests that the presence of LVI in prostate cancer is indicative of unfavorable prognosis. However, debate continues on the role of LVI as an independent prognosticator.

**TABLE 1**
**Characteristics of Cases with LVI**

| Case # | Age | Preop PSA | Gleason Score | EPE | SVI | CS | pT | LN met | Relapse | Time to Progress | Distant met |
|---|---|---|---|---|---|---|---|---|---|---|---|
| 1 | 60 | 13.7 | 7 | Present | No | (–) | 3a | Present | Yes | 36 | Absent |
| 2 | 60 | 41.5 | 7 | Present | Yes | (+) | 3b | Present | Yes | 26 | Absent |
| 3 | 71 | 7.2 | 8 | Present | Yes | (+) | 3b | Absent | Yes | 29 | Present |
| 4 | 51 | 8.9 | 9 | Present | Yes | (+) | 3b | Absent | Yes | 3 | Absent |
| 5 | 62 | 25.0 | 9 | Present | No | (+) | 3a | Absent | Yes | 35 | Absent |
| 6 | 69 | 24.1 | 9 | Present | Yes | (+) | 3b | Present | Yes | 24 | Absent |
| 7 | 65 | 28.0 | 7 | Present | No | (–) | 3a | Absent | Yes | 31 | Absent |
| 8 | 59 | 19.4 | 7 | Present | Yes | (+) | 3b | Absent | Yes | 84 | Absent |
| 9 | 72 | 11.0 | 7 | Present | Yes | (+) | 3b | Present | Yes | 21 | Absent |
| 10* | 60 | 8.2 | 7 | Present | Yes | (+) | 3b | Absent | NA | NA | NA |
| 11 | 67 | 8.5 | 7 | Present | No | (–) | 3a | Absent | No | 57 | Absent |

* Clinical follow-up data not available for this case. NA, not applicable.

**TABLE 2**
**Pathological Parameters and PSA Relapse According to Vascular Invasion**

| Variables | | Vascular Invasion | | |
|---|---|---|---|---|
| | | ***Absent*** **n (%)** | ***Present*** **n (%)** | ***p*** **Value** |
| Gleason score | 6 | 18 (100%) | 0 (0%) | 0.026 |
| | *7* | 30 (81.1%) | 7 (18.9%) | |
| | *>8* | 12 (75%) | 4 (25%) | |
| Extraprostatic extension | *Absent* | 23 (100%) | 0 (0%) | 0.002 |
| | *Present* | 36 (76%) | 11 (24%) | |
| Seminal vesicle invasion | *Absent* | 49 (91.5%) | 4 (7.5%) | 0.002 |
| | *Present* | 11 (61%) | 7 (39%) | |
| Positive surgical margins | *Absent* | 35 (92%) | 3 (8%) | 0.048 |
| | *Present* | 24 (75%) | 8 (25%) | |
| Pathological stage | pT2 | 23 (100%) | 0 (0%) | 0,001 |
| | pT3a | 25 (83.3%) | 5 (16.7%) | |
| | pT3b | 11 (61.1%) | 7 (38.9%) | |
| Lymph node metastasis | *Absent* | 57 (89%) | 7 (11%) | 0.001 |
| | *Present* | 1 (20%) | 4 (75%) | |
| PSA relapse | *Absent* | 44 (97.7%) | 1 (2.3%) | 0.001 |
| | *Present* | 13 (59%) | 9 (41%) | |

*Note:* n, number of cases evaluable.

Findings in our clinically localized prostate cancer cohort indicate that LVI has a positive correlation with Gleason score, extraprostatic extension, seminal vesicle invasion, positive surgical margins, and pathological stage, in addition to lymph node metastasis. All patients with LVI have Gleason score 7 or higher, and pathological stage of pT3a or T3b, with 64% seminal vesicle invasion. Seventy-three percent

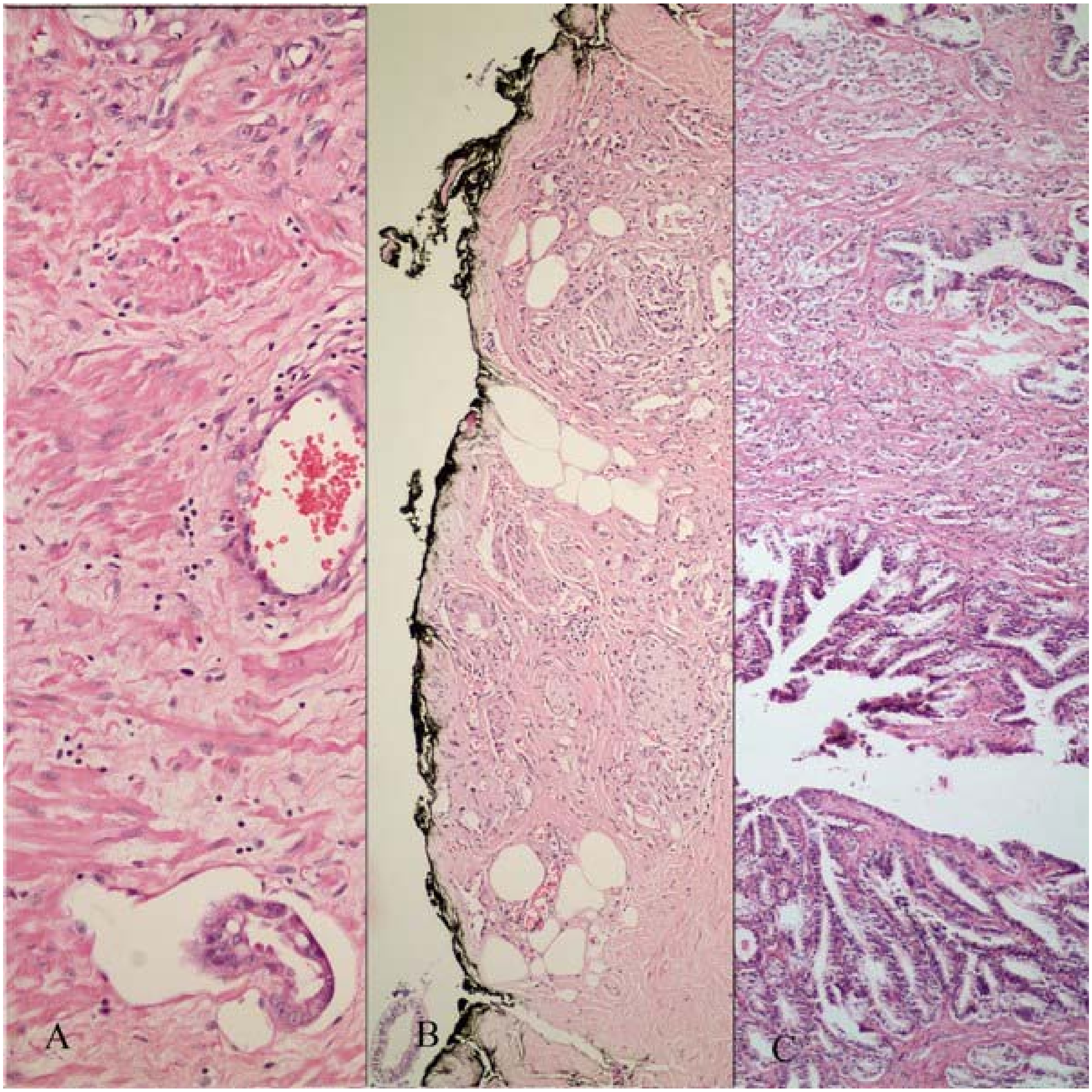

**FIGURE 2.** A neoplastic gland in a vascular space (A). The same tumor showing extraprostatic extension with positive surgical margins (B) and seminal vesicle invasion (C). (A: H-E ×400, B: H-E ×200; C: H-E ×200).

reveal positive surgical margins. Likewise, Loeb et al. reported significant association between LVI and tumor grade, tumor stage, tumor volume, and lymph node metastasis[16]. They additionally demonstrated statistically significant differences in rates of biochemical progression and 7-year PFS between patients with and without LVI. However, they could not confirm these findings in multivariate analysis. Their incidence of LVI is low (7%). An association between LVI and adverse outcomes, such as distant metastases, death, shorter PSA doubling time before salvage radiotherapy, and biochemical progression after salvage radiotherapy, has also been shown by Shariat et al. on univariate analysis[14]. Again, they have not observed LVI as an independent predictor of biochemical progression on multivariant analysis.

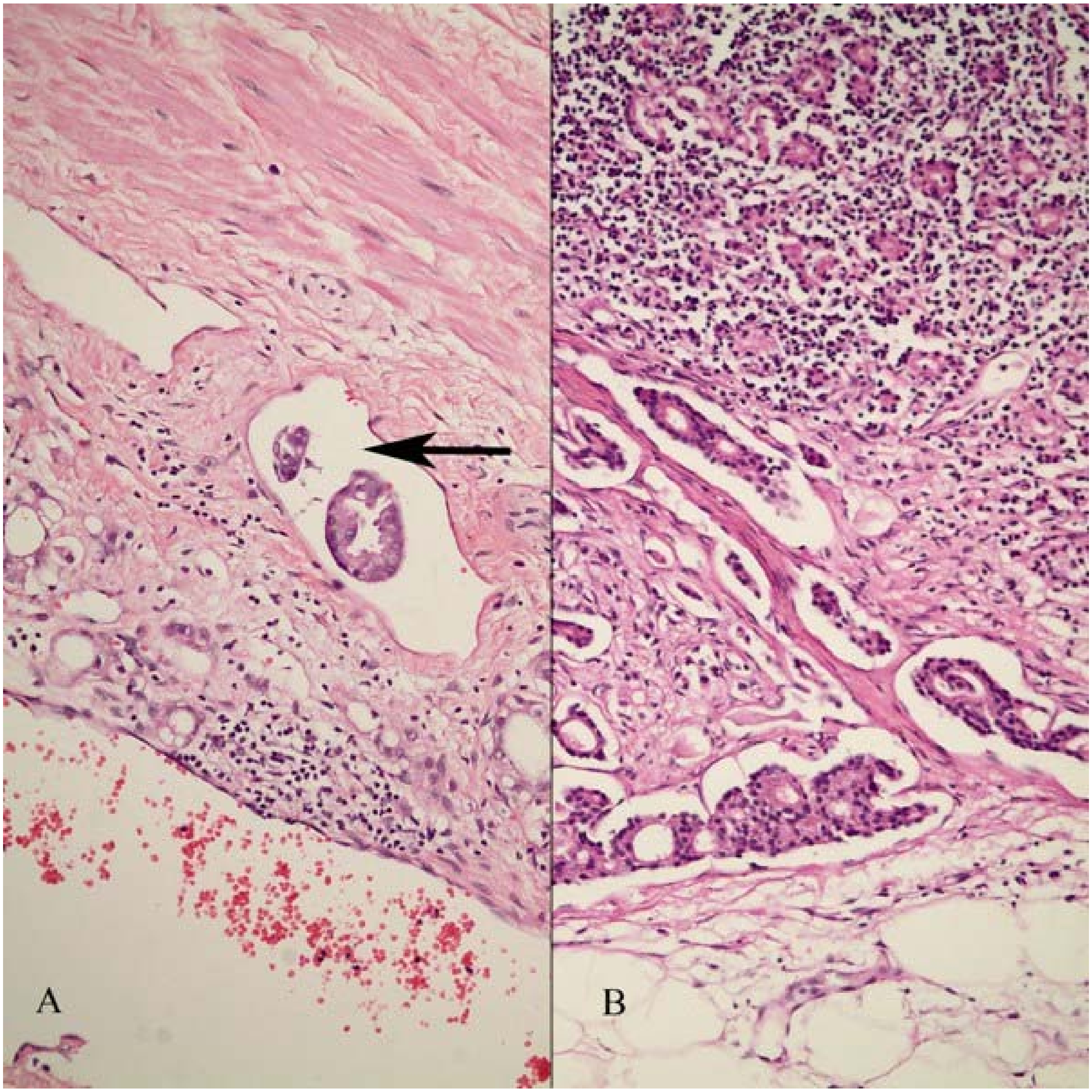

**FIGURE 3.** (A) LVI adjacent to a high-grade prostate carcinoma (H-E ×400). (B) Lymph node metastasis from the same tumor (H-E ×400).

Results of the studies that assess the prognostic value of a marker related to pathology depend highly on the criteria used to define the marker and on the selection of cohort. Variations in specimen handling by different laboratories and interobserver variability between pathologists further complicate the issue and lead to irreproducibility. Although case number can be considered limited, re-evaluation of pathology slides by a single pathologist who is expert in the field of genitourinary pathology, application of immunohistochemistry on equivocal or difficult conditions for confirmation of LVI, and relatively long mean follow-up terms are the features that support reliability in the present study.

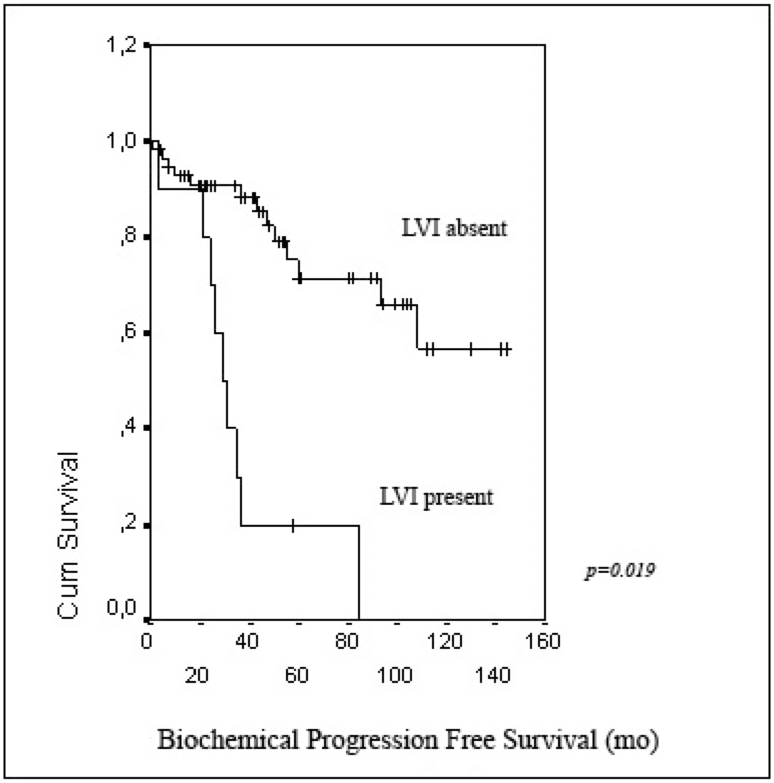

**FIGURE 4.** Biochemical PFS curves of the patients in regard to LVI.

In addition to a close relationship with known markers of poor prognosis, the high rate of positive LVI strongly correlates with increased biochemical recurrence in our series. On multivariate analysis, LVI and Gleason score values are independent, high-risk factors for biochemical failure. So LVI, as well as tumor grade, appears as predictive of PSA relapse in clinically localized prostate cancer patients who have RP. Survival analysis with Kaplan-Meier curves points to worse prognostic indication by LVI. We have detected a significant difference in biochemical PFS between patients with and without LVI. As expected, pathological stage and Gleason score are the other significant effectors of PSA relapse-free survival. Similarly, Babaian et al. found that pathologic category and LVI are the only significant independent predictors of progression in 265 RP patients[11]. Herman et al. studied stage pT3N0 disease, and observed that LVI is a significant predictor of progression on both univariate and multivariate analyses[8]. Five-year biochemical-free survival has been reported as 87.3% for patients with no LVI and 38.3% with LVI on the RP specimens ($p < 0.001$) by May et al.[17].

Consequently, RP specimens should be routinely evaluated for the presence of LVI. The clinical use of multivariate nomograms has provided a more accurate and relevant individualized prediction of survival for improved patient counseling and treatment. LVI is a pathological variable that might help clinicians to subclassify patients with an adverse risk of tumor progression and can be included in postoperative nomograms with other histological variables to select the group that might benefit from adjuvant therapies.

LVI by tumor cells is histologically evident in approximately 15.5% of prostatic carcinomas and is present in close to 10% of cases with pathologically negative lymph nodes in our series consisting of clinically localized cases. LVI is independently indicative of unfavorable prognosis in the prostate cancer as manifested by increased PSA failure and reduced biochemical PFS. It is for this reason that LVI should be routinely included in the evaluation and reporting of all prostate cancers.
